# Associations of Axial Length With Photoreceptor Mosaic and Retinal Nerve Fiber Bundle Microstructure Assessed by AO-SLO in Children

**DOI:** 10.1167/iovs.67.12.40

**Published:** 2026-10-21

**Authors:** Fengsheng Wang, Jiayi Li, Yue Zhao, Lushu Chen, Huiying Zhang, Qiuyang Zhang, Jin Yao

**Affiliations:** 1 The Affiliated Eye Hospital, Nanjing Medical University, Nanjing, People's Republic of China; 2 The Fourth School of Clinical Medicine, Nanjing Medical University, Nanjing, People's Republic of China

**Keywords:** children, myopia, adaptive optics scanning laser ophthalmoscopy (AO-SLO), nerve fibers, low-reflectivity cones

## Abstract

**Purpose:**

The purpose of this study was to characterize the microstructural remodeling of the photoreceptor mosaic and nerve fiber bundles during the progression of axial myopia in children, adaptive optics scanning laser ophthalmoscopy (AO-SLO) was used, to analyze their correlation with axial elongation, and to explore retinal microstructural changes to elucidate the pathogenesis and identify biomarkers of early myopia.

**Methods:**

High-resolution retinal imaging was performed using an AO-SLO system. Data on photoreceptor cells and nerve fibers were collected from 126 eyes of 126 children aged 7 to 16 years (11.52 ± 1.94). Statistical analysis was subsequently conducted to obtain parameters such as photoreceptor cell packing density (PCPD), cell spacing, dark cell ratio, nerve fiber thickness, and curvature.

**Results:**

Alterations in photoreceptor structure were observed across the temporal, nasal, superior, and inferior parafoveal regions. With increasing axial length (AL), photoreceptor cone density progressively declined and the inter-cellular spacing widened, with the most pronounced changes located in the temporal quadrant. In contrast, cellular regularity and mosaic dispersion remained largely preserved, suggesting no overt disruption of the packing geometry. In addition, the proportion of low-reflective (“dark”) photoreceptors increased, and the width of retinal nerve fiber bundles was reduced, whereas their curvature was not significantly affected.

**Conclusions:**

In summary, the series of cellular-level remodeling events accompanying axial elongation reveal a dynamic, coordinated interplay between retinal microstructure and ocular axial growth. These findings provide a new perspective for understanding the pathogenesis of myopia. Moreover, AO-SLO, as a noninvasive cellular-resolution imaging modality, demonstrates substantial potential for risk stratification and clinical screening of myopia progression.

Myopia, particularly in the pediatric population, has emerged as a major global public health challenge. In East Asian countries, more than half of children and adolescents are affected, among whom approximately 10% to 20% progress to high myopia. By 2050, the worldwide prevalence of myopia is projected to approach 49.8%.^1^^–^^3^ China currently reports the highest prevalence of childhood and adolescent myopia globally, with a continuing upward trend.^4^^,^^5^ The progression of myopia is characterized not only by increasing refractive error but also by excessive axial elongation, which substantially elevates the risk of irreversible vision-threatening complications such as retinal detachment, myopic maculopathy, and open-angle glaucoma.^6^^–^^8^

Axial elongation is widely recognized as a fundamental structural hallmark of myopia development. Currently, the prediction and diagnosis of childhood myopia primarily rely on measurements of refractive error and ocular axial length (AL), reflecting both functional and anatomic alterations associated with myopic progression. However, these conventional indicators are insufficient to capture pathological alterations at the level of retinal microarchitecture, and their sensitivity and predictive value remain limited.^9^^,^^10^ Meanwhile, studies focusing on cellular-scale retinal changes during the process of axial elongation are still scarce, and definitive evidence supporting their use as novel predictive or diagnostic biomarkers is lacking. Although traditional ophthalmic imaging modalities, such as fundus photography and optical coherence tomography (OCT), can visualize gross anatomic features. This limitation makes it difficult to accurately and dynamically assess retinal microstructures, particularly photoreceptor cells in vivo.^11^^,^^12^

The advent of adaptive optics scanning laser ophthalmoscopy (AO-SLO) has effectively overcome the resolution limitations of traditional retinal imaging techniques, enabling in vivo visualization at a submicron scale.^13^^,^^14^ This technology provides a powerful platform for directly assessing microstructural alterations of the retina during myopia progression and following therapeutic intervention.^15^^,^^16^ Recent studies have utilized AO-SLO to characterize ultrastructural changes in photoreceptors among children undergoing repeated long-term low-level red-light (RLRL) treatment, supplying critical evidence for both the safety and efficacy of this approach.^16^ In addition, AO-SLO is increasingly being incorporated into disease risk stratification and progression monitoring, underscoring its significant translational and clinical potential.^13^^,^^17^

Therefore, the application of AO-SLO technology to the investigation of pediatric myopia offers substantial scientific and clinical value. AO-SLO enables cellular-level visualization of retinal pathology, thereby facilitating a deeper understanding of the mechanisms underlying myopia onset and progression and supporting the development of novel predictive biomarkers and therapeutic targets. On the other hand, it provides an unprecedented level of precision for evaluating both the efficacy and microstructural safety of various myopia control interventions.^16^^,^^18^^–^^20^

Although AO-SLO has been applied to quantify photoreceptors in the peripheral retina of patients with myopia, prior studies have largely been limited to adult populations or small sample sizes.^16^^,^^19^^,^^21^ Consequently, there remains a lack of large-scale, cell-level evidence in children, as well as a paucity of integrated analyses combining photoreceptor and nerve fiber characteristics. Accordingly, the present study aims to further characterize photoreceptor and nerve fiber features in children with axial elongation using AO-SLO, and to investigate their structural alterations and inter-relationships during disease progression. Through this approach, we endeavor to investigate potential mechanisms of childhood myopia and hope that our findings may contribute to a theoretical and technical framework for early prediction and precision intervention.

## Materials and Methods

### Ethical Approval

The study was conducted in accordance with the principles of the Helsinki Declaration and was approved by the Institutional Ethics Committee of the Eye Hospital of Nanjing Medical University (No. 2023001). Written informed consent was obtained from all participants prior to enrollment.

### Participants

A total of 126 healthy children aged 7 to 16.54 years (11.52 ± 1.94) were recruited from the Eye Hospital of Nanjing Medical University. All participants underwent comprehensive ophthalmic examinations, including slit-lamp biomicroscopy, fundus evaluation, best-corrected visual acuity (BCVA), AL (measured with IOL Master), refraction, and AO-SLO imaging. Inclusion criteria required BCVA ≥ 20/20, normal intraocular pressure, and astigmatism < 2.00 diopters (D). Exclusion criteria included retinal disease, systemic disorders, or poor image quality. AL ranged from 22.18 to 27.30 mm (24.45 ± 1.03). Based on spherical equivalent refraction (SE), eyes were divided into four groups according to the quartiles of refractive error (−2.75, −1.5, and −0.75), with boundary values assigned using half‑open intervals (lower and upper) to ensure that each subject was classified into a single subgroup: group A, −0.75 D < SE ≤ 0.50 D; group B, −1.50 D < SE ≤ −0.75 D; group C, −2.75 D < SE ≤ −1.50 D; and group D, −6.0 D ≤ SE < −2.75 D.

### Apparatus

As described previously,^22^ photoreceptor images were acquired using the AO-SLO system (Mona IIa; Robotrak Technology Co., Ltd.). The AO-SLO imaging module and the wavefront sensor were driven by a common 840 nm light source (SLD, central wavelength 840 nm, bandwidth 40 nm), providing a retinal field of view of either 2.4 degrees × 2.4 degrees (approximately 700 µm × 700 µm) or 5 degrees × 5 degrees (approximately 1440 µm × 1440 µm). Prior to detection, a confocal pinhole with a diameter approximately twice the Airy disk was positioned in front of the avalanche photodiode detector. Ocular aberrations were corrected in real time through a high-speed deformable mirror in combination with a custom Shack–Hartmann wavefront sensor. The imaging power at the pupil was maintained below 600 µW, well within the safety limits specified by the American National Standards Institute (ANSI). To compensate for individual variations in ocular magnification, all image measurements were calibrated using the Littmann–Bennett correction formula, with AL values measured for each eye separately.^23^ Image quality was assessed using a confidence metric, which was derived from two factors: the prediction probability output by the automated cell recognition artificial intelligence (AI) model, and the clarity of cell borders discernible in the original images.

### Procedures

For photoreceptor imaging, a field of view of 2.4 degrees × 2.4 degrees (approximately 700 µm × 700 µm) was acquired. For each participant, photoreceptor mosaics were captured at four quadrants (temporal, nasal, superior, and inferior). Subsequently, regions located 1.2 degrees from the foveal center in each quadrant were selected for quantitative analysis (Fig. 1). All images were centered on the fovea and aligned along the meridians, with care taken to avoid retinal vessels or other artifacts that could compromise image quality. The sampling offset was maintained below 0.1 degrees, and each site was imaged five times to obtain repeated measurements. All acquisitions were performed by a single ophthalmologist specialized in retinal functional imaging, ensuring methodological consistency.

**Figure 1.**
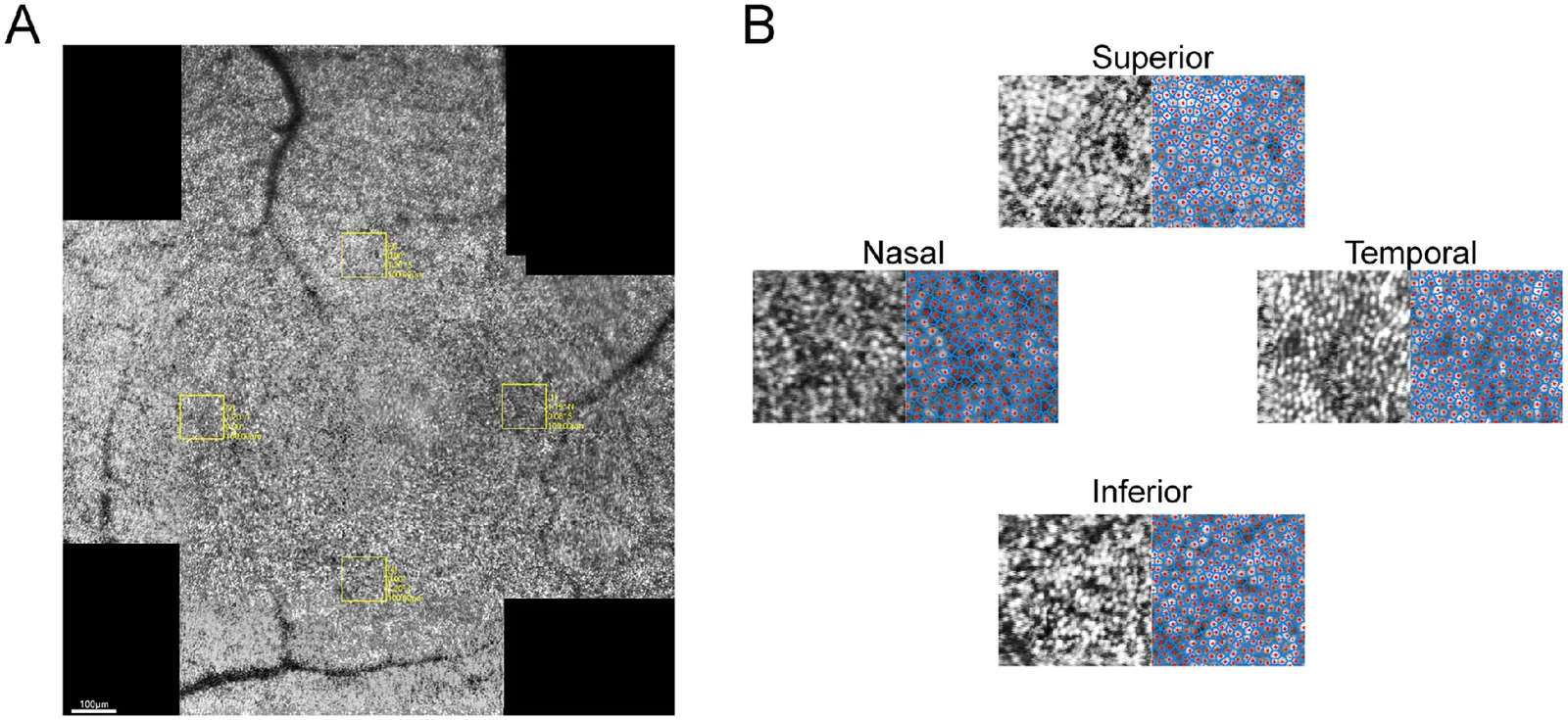
Representative example of photoreceptor imaging with AO-SLO. (**A**) Composite image spanning from the foveal center to the periphery, with *yellow boxes* marking regions at 1.2 degrees eccentricity (temporal, nasal, superior, and inferior). (**B**) High-resolution view from a boxed region in **A**, showing the photoreceptor mosaic and corresponding segmentation map generated by analysis software.

Quantitative analysis was conducted using adaptive optics-based evaluation of photoreceptor cell packing density (PCPD), cell spacing, as well as metrics of dispersion and regularity. Dispersion, reflecting the variability of intercellular spacing, was calculated as the ratio of the standard deviation (SD) to the mean of the nearest-neighbor distances. Regularity, reflecting the uniformity of cone mosaic organization, was determined by calculating the proportion of cells with four, five, six, or seven neighbors relative to the total cell count, with values approaching six neighbors indicating a more regular cellular arrangement.^24^^,^^25^ After segmentation of photoreceptor cells, a semi‑automated ImageJ software script was used to quantify the grayscale intensity of all cells within the region‑of‑interest (ROI) mosaics. Cells with an intensity below 50% of the image-specific mean grayscale value of the corresponding image were defined as dark cells and subjected to subsequent statistical analyses.^26^

For nerve fiber analysis, images were acquired from the superotemporal retina at approximately 2.5 degrees eccentricity, covering a 5 degrees × 5 degrees field (approximately 1440 µm × 1440 µm). Within this region, the width and curvature of nerve fibers were quantified. All visible nerve fiber widths within the selected region were measured and quantified using ImageJ software and calculating their mean value. Curvature was assessed using the Kappa plugin in ImageJ software (Fig. 2). All measurements were performed by a single masked examiner to ensure consistency.

**Figure 2.**
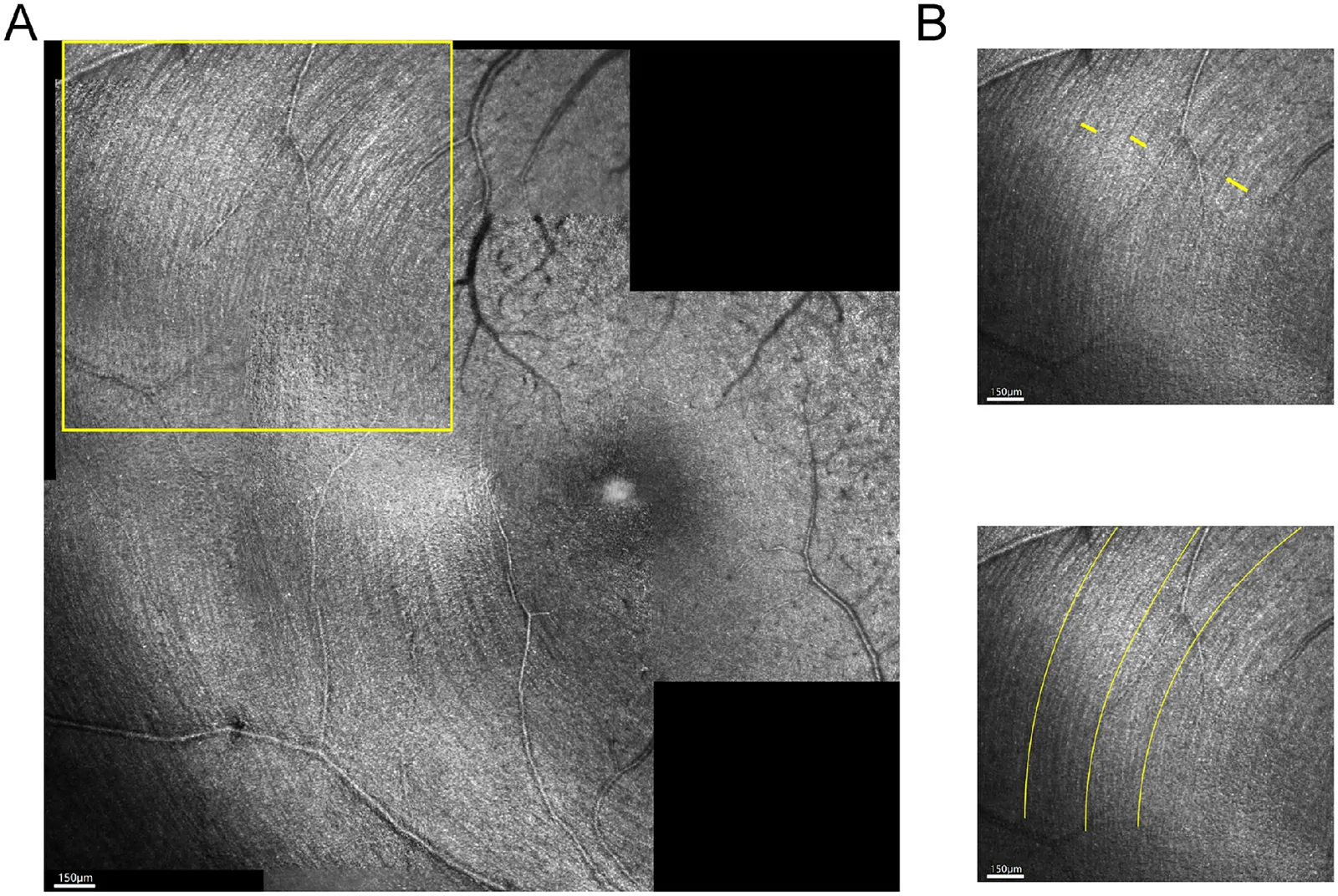
Representative example of nerve fiber imaging with AO-SLO. (**A**) Composite image extending from the fovea toward the temporal retina, with the *yellow box* indicating the selected 5 degrees × 5 degrees region (approximately 1440 µm × 1440 µm). (**B**) Magnified AO-SLO image from the boxed region in panel **A**. Nerve fiber width and curvature were quantified by sampling fibers within this field.

To ensure the reliability of the experimental findings, all images with a confidence value below 50% were excluded, and the confidence score was further adjusted as a covariate in the multiple linear regression analysis.

### Statistical Analysis

Data are presented as mean ± SD. All statistical analyses and graphical presentations were performed using SPSS version 26 (IBM Corp., Armonk, NY, USA) and GraphPad Prism version 10.1.2 (GraphPad Software, San Diego, CA, USA). A two-tailed *P* value < 0.05 was considered statistically significant. Due to missing reports, AL data were unavailable for 11 subjects (8.7%). Additionally, due to poor image quality, six cases (4.8%) of AO-SLO photoreceptor imaging data were excluded. AL data were addressed using multiple imputation, and the median of five imputed datasets was adopted for subsequent analyses. Comparisons among multiple groups were conducted using one-way analysis of variance (ANOVA). Categorical variables were analyzed with the chi-square test. Associations between variables were further assessed through linear regression analysis. Normality tests, Pearson correlation analysis, and multivariable regression were additionally performed.

## Results

This study included 126 eyes from 126 healthy children (54 male and 72 female children), aged 7 to 16.54 years (11.52 ± 1.94). Refractive error, expressed as SE, ranged from –6.00 D to +0.50 D (–1.86 ± 1.50 D), whereas AL ranged from 22.18 to 27.13 mm (24.45 ± 1.03 mm). Considering that myopia in children predominantly falls within the mild-to-moderate range, participants were stratified into 4 subgroups according to SE: group A, −0.75 D < SE ≤ 0.50 D (*n* = 25); group B, −1.50 D < SE ≤ −0.75 D (*n* = 31); group C, −2.75 D < SE ≤ −1.50 D (*n* = 38); and group D, −6.0 D ≤ SE < −2.75 D (*n* = 32). Baseline demographic and ocular characteristics are provided in Table 1. SE showed no significant association with sex or laterality but correlated significantly with age, astigmatism, and AL. Linear regression confirmed a strong negative relationship between AL and SE (Fig. 3; *P* < 0.0001, *R*² = 0.4544).

**Table 1.** Baseline Ocular and Demographic Characteristics of the Study Group

Group	*N*	Sex, M/F	Age	Eye Difference, OD/OS	SE, D	Cyl, D	AL, mm
A	25	12/13	11.03 ± 1.74	14/11	−0.06 ± 0.35	−0.39 ± 0.42	23.53 ± 0.80
B	31	11/20	11.28 ± 1.85	14/17	−1.06 ± 0.21	−0.35 ± 0.53	24.22 ± 0.68
C	38	14/24	11.08 ± 1.59	18/20	−1.93 ± 0.35	−0.67 ± 0.41	24.47 ± 0.79
D	32	17/15	12.65 ± 2.17	20/12	−3.98 ± 0.93	−0.83 ± 0.41	25.38 ± 0.98
*F*/χ2		χ2 = 2.897	*F* = 5.497	χ2 = 2.475	*F* = 278.571	*F* = 8.198	*F* = 25.294
*P* value		0.408	0.001	0.480	<0.001	<0.001	<0.001

**Figure 3.**
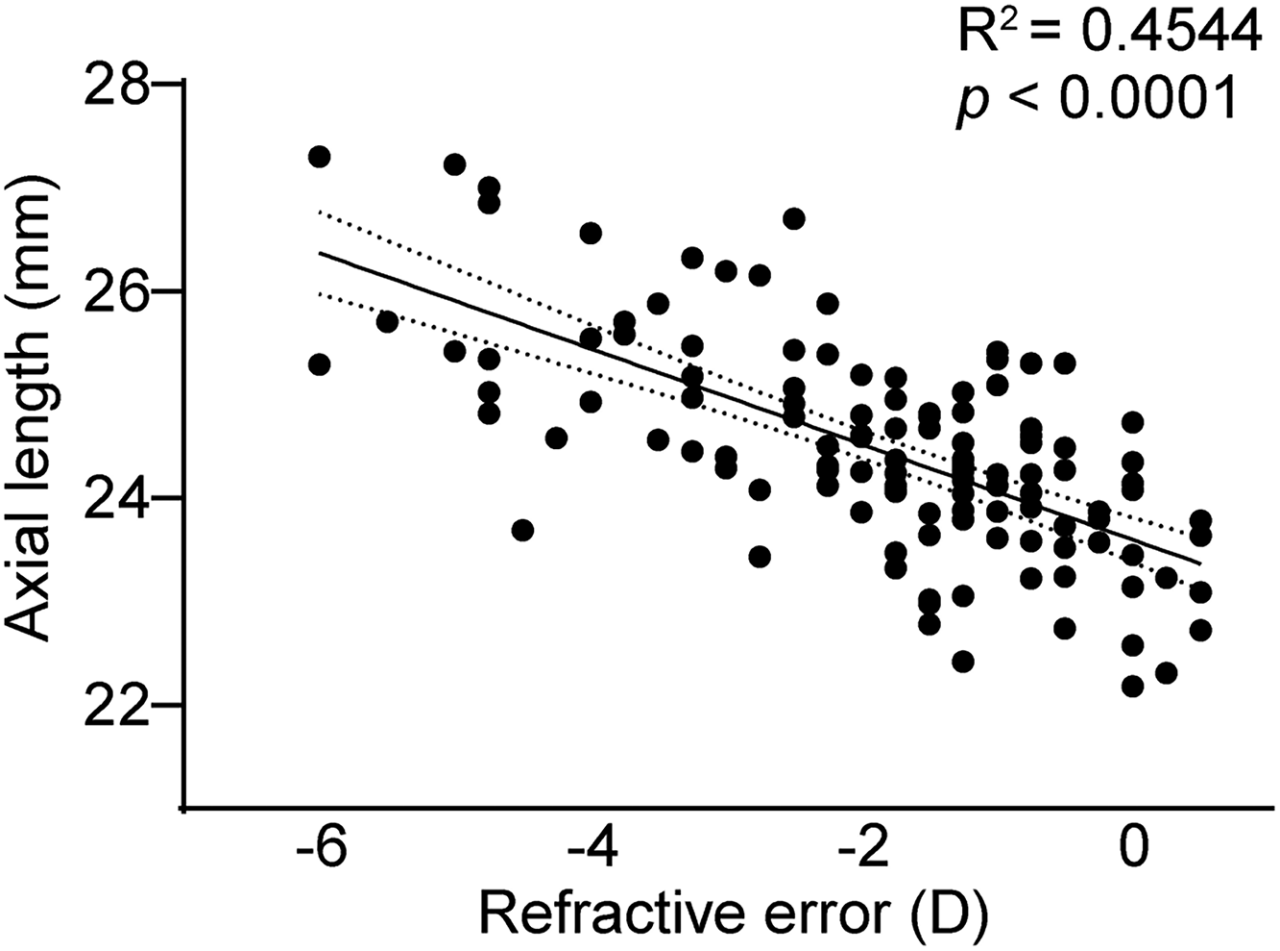
Association between AL and refractive error based on linear regression.

The ROIs for statistical analysis were extracted from the composite images at 1.2 degrees eccentricity from the foveal center, encompassing the temporal, nasal, superior, and inferior quadrants. Across these 4 locations, photoreceptor cell density ranged from 19,852.32 to 55,868.22 cells/mm² (34,761.42 ± 5661.35), whereas the mean intercellular spacing varied between 3.71 and 5.67 µm (4.51 ± 0.29). Grouped by refractive error, parameters including PCPD, cell spacing, nearest-neighbor counts, mosaic regularity, and dispersion were systematically analyzed. The results demonstrated that, compared with the emmetropic group, increasing myopic refractive error was associated with a progressive reduction in PCPD and an enlargement of cell spacing (Table 2). Detailed statistical outcomes are presented in Supplementary Table S1.

**Table 2.** Summary of AO-SLO-Derived Imaging Parameter

	Parameters	Group A	Group B	Group C	Group D	*F*	*P* Value
Temporal	PCPD, mm^2^	38,015.04 ± 5,262.87	35,625.68 ± 5,650.16	34,789.40 ± 5,204.59	31,349.04 ± 4,823.28	8.027	<0.001
	Cell spacing, µm	4.33 ± 0.28	4.48 ± 0.28	4.52 ± 0.26	4.67 ± 0.27	7.629	<0.001
Nasal	PCPD, mm^2^	37,404.75 ± 4,187.33	36,836.57 ± 5,773.14	35,649.29 ± 6,698.26	32,538.82 ± 8,462.83	3.280	0.023
	Cell spacing, µm	4.35 ± 0.22	4.42 ± 0.27	4.50 ± 0.27	4.65 ± 0.36	5.914	0.001
Superior	PCPD, mm^2^	36,887.69 ± 4,094.67	33,689.26 ± 3,627.25	33,660.69 ± 4,254.17	31,591.74 ± 3,335.26	8.878	<0.001
	Cell spacing, µm	4.47 ± 0.27	4.66 ± 0.27	4.63 ± 0.21	4.75 ± 0.24	6.577	<0.001
Inferior	PCPD, mm^2^	37,026.30 ± 4,918.90	35,511.45 ± 5,956.32	34,514.57 ± 5,470.57	32,012.48 ± 4,059.36	4.828	0.003
	Cell spacing, µm	4.40 ± 0.37	4.46 ± 0.28	4.56 ± 0.33	4.68 ± 0.28	4.538	0.005

Results indicated that PCPD and cell spacing were significantly correlated with refractive error, whereas measures of regularity and dispersion showed no substantial differences. These findings suggest that myopia progression may be accompanied by reduced photoreceptor density and increased spacing, but not by disruption of the overall mosaic organization or deterioration of cellular regularity. These quadrant-specific differences, particularly the more pronounced alterations in the temporal and superior regions (Fig. 4), may reflect localized susceptibility of the retina to asymmetric axial elongation during myopia progression, providing important clues for understanding the underlying mechanisms.

**Figure 4.**
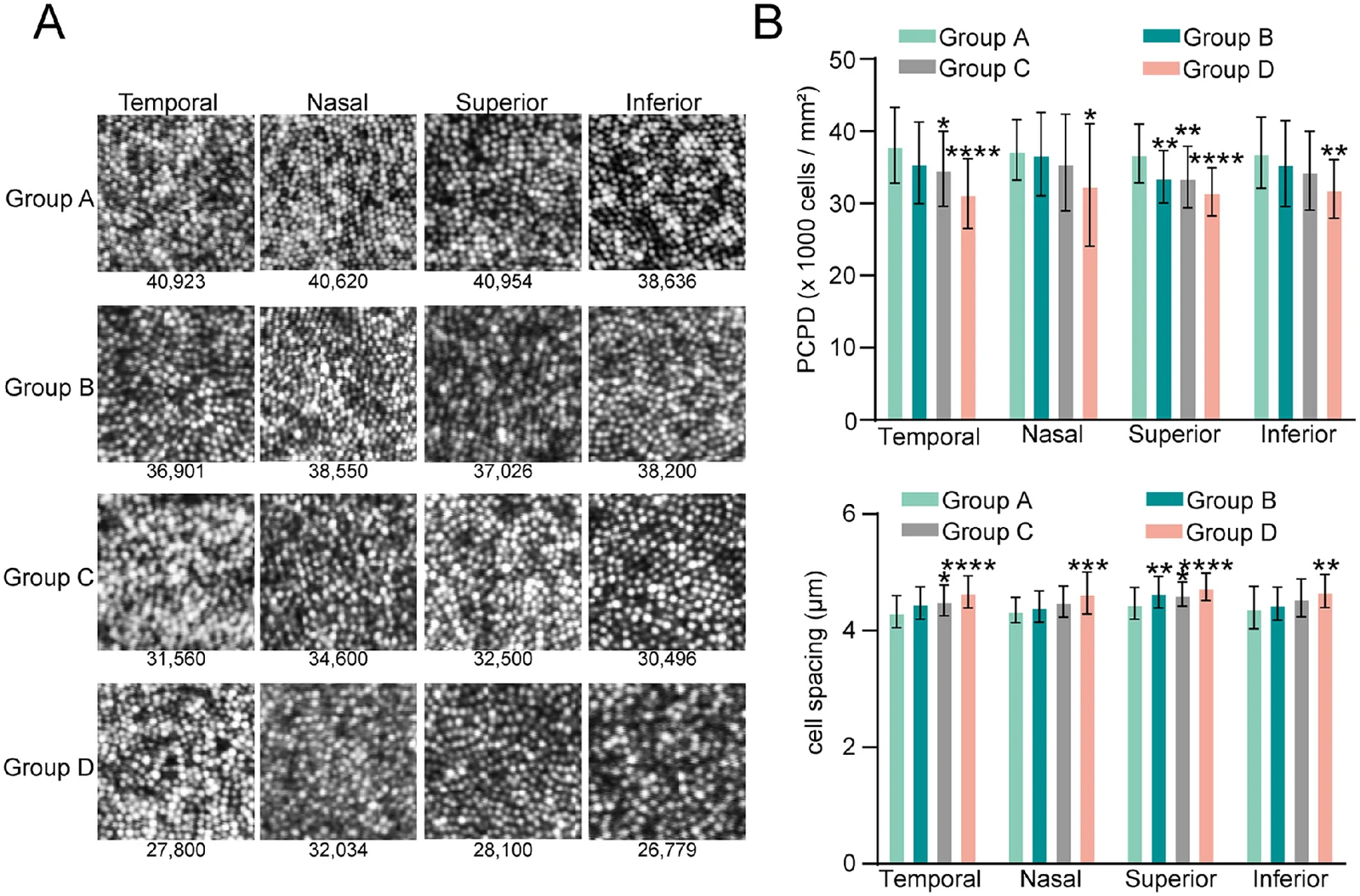
Alterations in photoreceptor density and spacing assessed by AO-SLO. (**A**) Representative AO-SLO images of photoreceptors within a 100 µm × 100 µm region across the four retinal quadrants. (**B**) Quantitative analysis of photoreceptor density and spacing. Comparisons were performed using one-way ANOVA, with all post hoc tests referenced to group A. *P < 0.05, **P < 0.01, ***P < 0.001, ****P < 0.0001.

During the study, we observed regional heterogeneity in PCPD and cell spacing across the four quadrants centered on the fovea. To further investigate the relationship between these microstructural parameters and myopia, linear regression analyses were performed between photoreceptor density/spacing and AL (Fig. 5). A significant association was observed between both parameters and AL, supporting the notion that axial elongation is associated with substantial retinal microstructural changes. Notably, cell spacing in the temporal quadrant showed the highest correlation with AL (*P* < 0.0001, *R*² = 0.4516).

**Figure 5.**
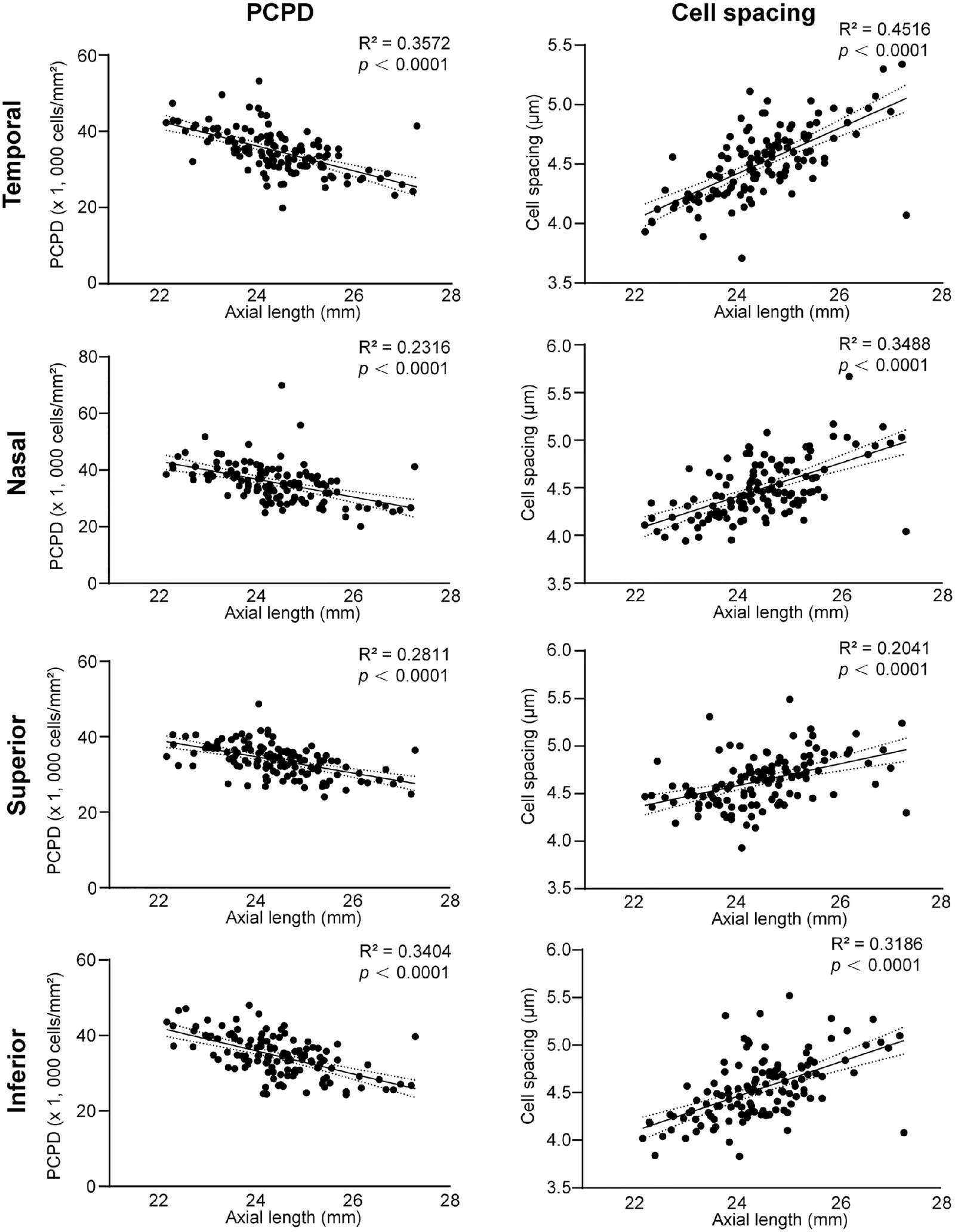
Linear regression analysis of AO-SLO-derived parameters with AL.

To investigate whether the effect of AL on photoreceptor cell density was independent of image quality (confidence), we performed a multiple linear regression model that included both AL and confidence as independent variables. After adjusting for confidence, AL remained significantly and negatively associated with temporal photoreceptor density (β = −3154, 95% confidence interval [CI] = −3864 to −2445, *P* < 0.0001), indicating that the effect of AL on photoreceptor density was independent of image quality (Supplementary Fig. S1, Supplementary Table S2). These findings imply that PCPD and cell spacing could serve as a potential auxiliary biomarker for the screening and early diagnosis of myopia.

To minimize confounding from age-related retinal maturation, a subgroup analysis was performed in an age-homogeneous group of 10-year-old participants (*n* = 26). Focusing on the temporal retina—the region most sensitive to AL in the primary analysis—we observed a consistent pattern: PCPD decreased with increasing AL, associated with a corresponding increase in cell spacing (Fig. 6A). Although residual age effects cannot be entirely excluded, the reproducibility of these trends within an age-controlled group suggests that the observed photoreceptor alterations are primarily attributable to pathological axial elongation rather than physiological maturation.

**Figure 6.**
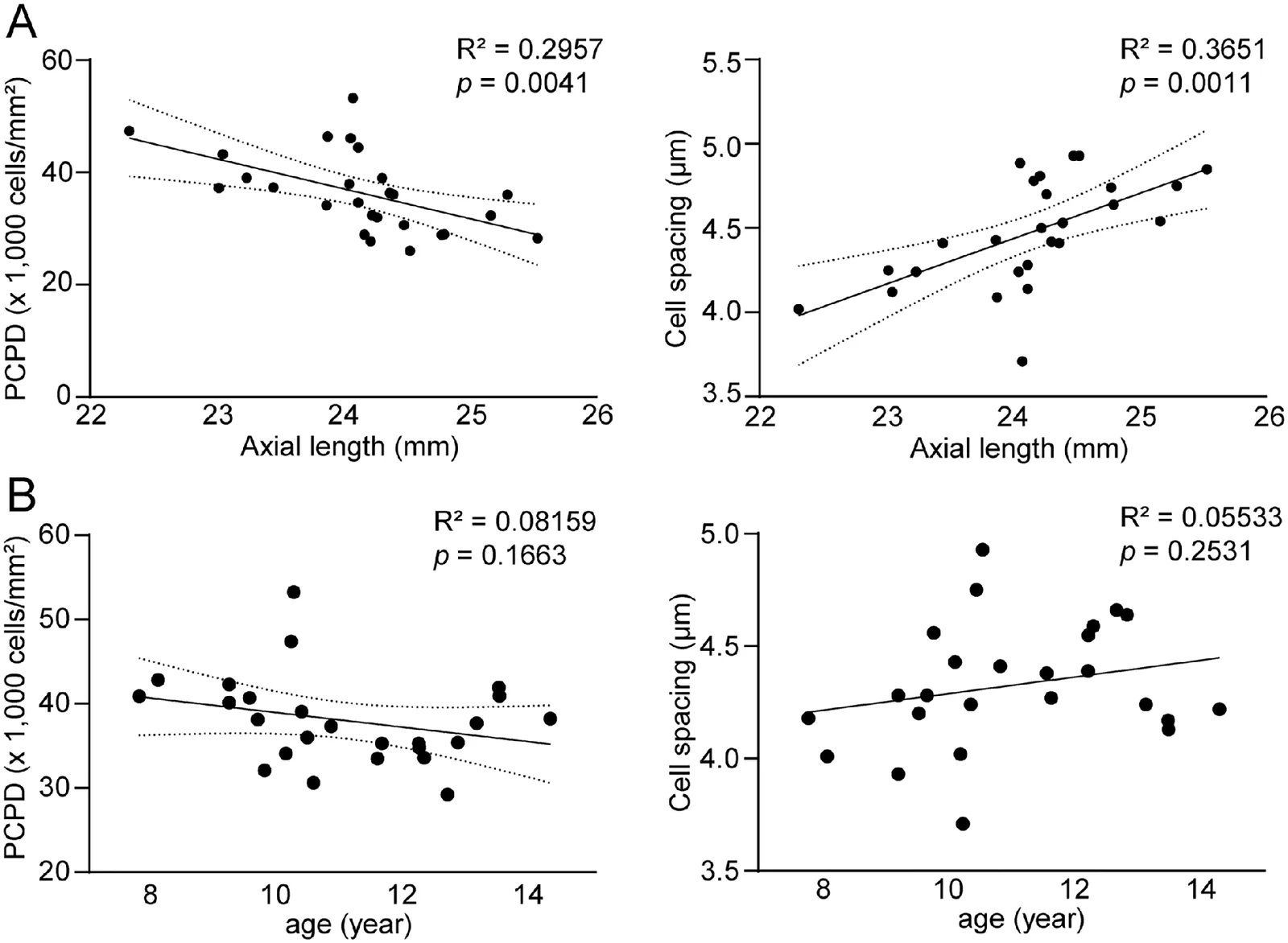
Linear regression plots illustrating the relationship between AL and photoreceptor packing density/cell spacing in 10-year-old and emmetropic children. (**A**) Linear regression plots illustrating the relationship between AL and photoreceptor packing density/cell spacing in 10-year-old children. (**B**) Linear regression plots illustrating the relationship between AL and photoreceptor packing density/cell spacing in emmetropic children.

In addition, an emmetropic control group (*n* = 25) was examined to assess age effects. Linear regression revealed no significant association between age and either PCPD (*P* = 0.1663) or spacing (*P* = 0.2531) in the temporal quadrant (Fig. 6B). This discrepancy from prior reports may be due to the limited sample size, reducing statistical power.^27^^,^^28^

Collectively, these findings indicate that axial elongation is associated with quantifiable microstructural disorganization of the photoreceptor mosaic, characterized by reduced PCPD and cell spacing. Such remodeling appears to reflect pathological rather than physiological processes and may precede functional decline, highlighting its potential as an early biomarker of myopia progression.

To distinguish whether the decline in PCPD arises primarily from morphological remodeling associated with axial elongation or from actual photoreceptor damage and loss, we quantified the proportion of “dark cells” within the temporal region of interest in each subject. These dark cells—defined as photoreceptors exhibiting significantly reduced reflectance—are considered potential indicators of early structural compromise or metabolic dysfunction.^29^^–^^31^ Quantitative analysis demonstrated that the proportion of dark cells increased progressively with the degree of myopia (Fig. 7). This association remained evident when image quality was controlled for (Supplementary Fig. S2A, Supplementary Table S3). Such a pattern may signify early photoreceptor stress or degeneration, and could also, in part, result from geometric distortion of the retinal plane caused by progressive axial elongation.

**Figure 7.**
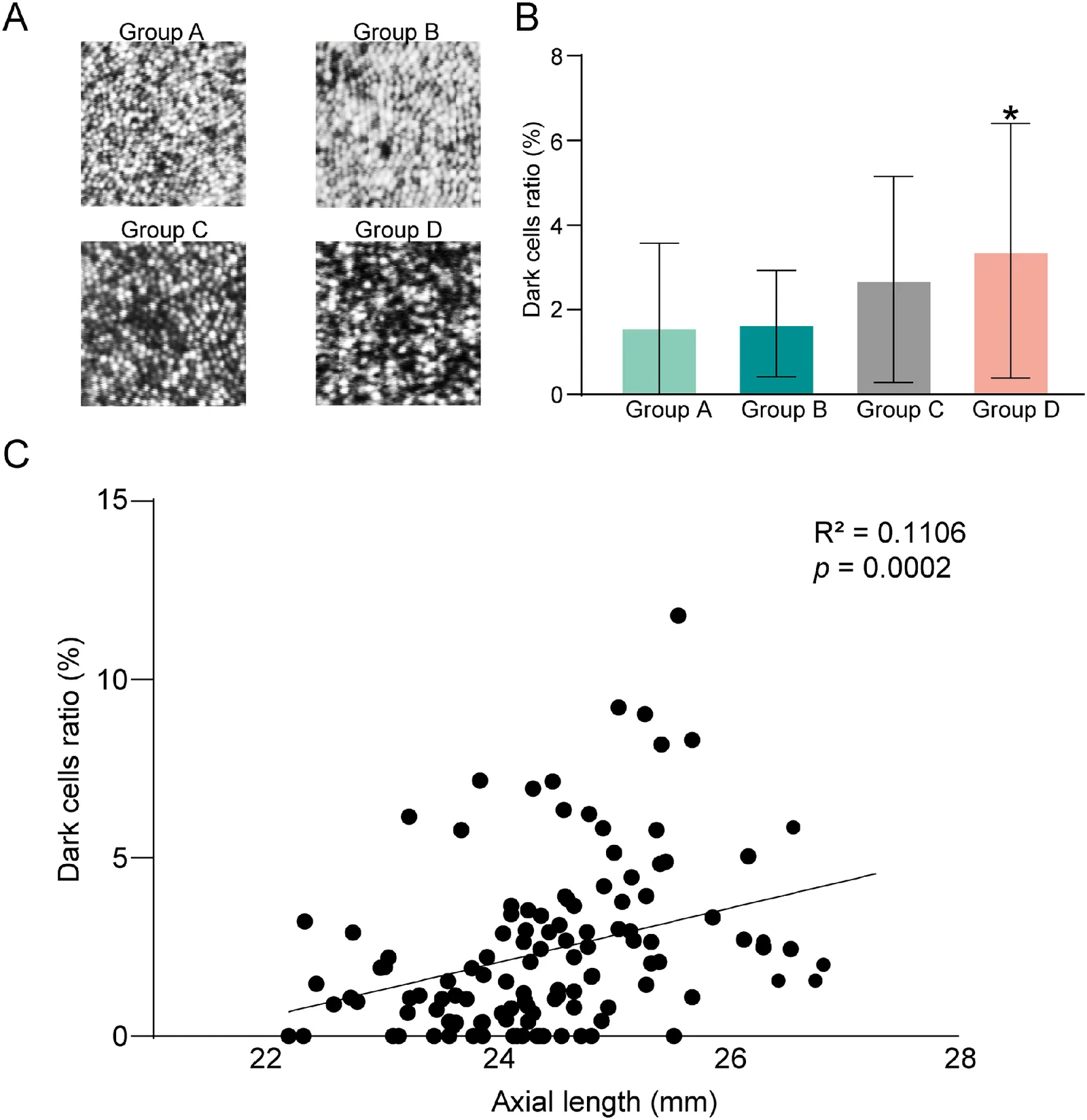
Quantification of dark photoreceptors in AO-SLO images. (**A**) Representative AO-SLO montages illustrating dark photoreceptor appearances across different refractive status groups. (**B**) Group-wise comparison of the proportion of dark photoreceptors within the temporal region of interest. (**C**) Linear regression analyses demonstrating the relationship between the proportion of dark cells and AL. Comparisons were performed using one-way ANOVA, with all post hoc tests referenced to group A. *P < 0.05.

Following the assessment of photoreceptor cells, we further investigated the potential involvement of nerve fibers in the progression of refractive error‑related ocular changes. The results demonstrated that with increasing refractive error, the curvature of nerve fibers remained largely unchanged (Fig. 8A), whereas nerve fiber width exhibited a significant decrease (Fig. 8B). A notable correlation between nerve fiber width and AL was also observed (Fig. 8C, Supplementary Table S4). To explore this phenomenon in greater depth, the relationship between nerve fiber width and the proportion of dark cells was examined. Multiple linear regression analysis indicated that the association between dark cells and nerve fiber width could be largely accounted for by variations in AL (Fig. 8D, Supplementary Table S5).

**Figure 8.**
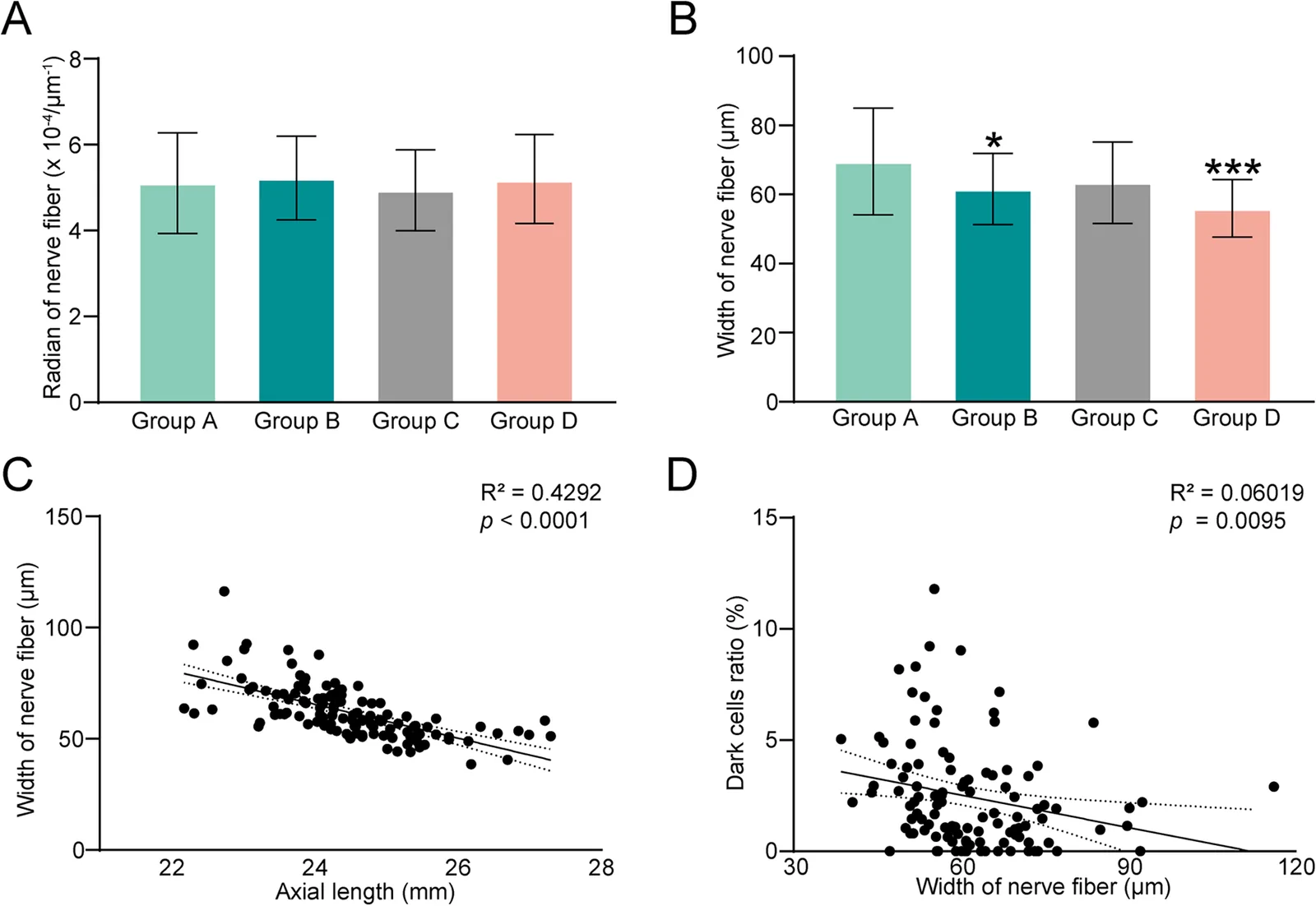
Quantitative analysis of retinal nerve fibers imaged with AO-SLO. (**A**) Comparison of nerve fiber radian across refractive error groups. (**B**) Comparison of nerve fiber width across refractive error groups. (**C**) Linear regression analysis of nerve fiber width and AL. (**D**) Linear regression analysis of nerve fiber width and dark cells ratio. One-way ANOVA was used for group comparisons, with post hoc analyses performed relative to group A. *P < 0.05, **P < 0.01, ***P< 0.001.

## Discussion

In this study, we used AO-SLO to obtain in vivo, high-resolution retinal microstructural data from 126 eyes of 126 children with refractive errors, which represents one of the earliest large-sample AO-SLO investigations in children, simultaneously evaluating both photoreceptor and nerve fiber characteristics. By sampling four quadrants (temporal, nasal, superior, and inferior) at 1.2 degrees eccentricity from the foveal center, we were able to visualize both cone photoreceptor mosaics and the nerve fiber layer at a cellular resolution rarely achievable in pediatric groups. Consistent with previous findings in adolescents and adults,^16^^,^^22^^,^^32^ we observed a progressive reduction in cone density and expansion of intercone spacing with increasing myopic severity. Importantly, this pattern was not uniformly distributed but was most prominent in the temporal quadrant, suggesting a regionally preferential susceptibility of this sector to myopia-associated retinal stretching.

In myopic eyes, structural alterations in the peripheral retina exhibit a pronounced spatial asymmetry, with changes in the temporal region occurring earlier than in other quadrants. This phenomenon likely arises from multiple inter-related mechanisms. First, at the optical level, relative peripheral hyperopia in the temporal retina has been proposed to play a dominant role in myopia progression. This refractive asymmetry subjects the temporal retina to stronger hyperopic defocus signals, which in turn stimulate active scleral elongation and choroidal thinning.^33^^,^^34^ Notably, choroidal thickness changes precede axial elongation in the development of myopia, and studies have shown that choroidal thickness decreases progressively from the nasal to the temporal side, with the temporal mid‑peripheral region exhibiting the thinnest choroid, thereby further exacerbating the structural vulnerability of this area.^35^^,^^36^ Concurrently, evidence indicates that the reduction in retinal thickness and vessel density is also most pronounced in the temporal quadrant.^37^ Second, at the biomechanical level, axial elongation does not occur symmetrically across the entire posterior pole, but rather preferentially expands anisotropically along the horizontal meridian,^38^^,^^39^ with early deformation primarily originating from the anterior sclera or equatorial sclera.^40^^,^^41^ This localized scleral remodeling preferentially transmits biomechanical tension to the temporal retina, accelerating photoreceptor cell displacement and local microstructural rearrangement. The combined effects of these optical and mechanical factors render the temporal macular region a sensitive site for myopia‑related cellular remodeling. Although direct histological validation is currently lacking, existing structural and imaging evidence strongly supports the identification of the temporal retina as a critical region for early monitoring and diagnosis in childhood myopia progression. To further clarify whether these changes were truly associated with AL rather than confounded by age, we performed subgroup analyses controlling for the parameters. After adjusting for this covariate, the association between AL and cone density/intercone spacing remained statistically significant. This indicates that myopia itself, rather than physiological maturation, is the dominant factor of early microstructural remodeling in this group. Interestingly, compared with adult group reported previously,^22^^,^^42^ children demonstrated a more pronounced photoreceptor response to AL progression, suggesting that the pediatric retina may be more biomechanically plastic — or, conversely, more vulnerable — during early ocular development.^43^^,^^44^

To investigate the specific impact of axial elongation on photoreceptor integrity, the proportion of photoreceptors exhibiting reduced reflectance was quantified, defined as dark cells. Our analysis revealed a significant positive correlation between the proportion of dark cells and AL, suggesting that the quantification of dark cells may serve as a potential biomarker for clinical diagnosis and disease monitoring in myopia. Furthermore, these findings offer a novel perspective for understanding the structural alterations accompanying myopic progression, thereby contributing to the broader exploration of myopia pathogenesis.^13^^,^^27^^,^^43^

These hyporeflective photoreceptors may reflect alterations in the optical coupling efficiency of the cells. To maximize light capture efficiency, photoreceptors are oriented toward the center of the pupil, with their inner and outer segments functioning as optical waveguides that direct light propagation. Changes in the angle of light incidence can affect capture and transmission efficiency.^45^^–^^47^ Axial elongation may alter the spatial arrangement of photoreceptors, particularly the tilt of outer segments relative to the incident light axis, thereby disrupting waveguiding properties and diminishing directional reflectance, ultimately manifesting as an increased proportion of dark cones. In addition, these low‑reflectance photoreceptors may also indicate structural or functional compromise—such as early outer segment disruption, metabolic stress, or incipient degenerative changes.^48^^,^^49^ Metabolic alterations in the mitochondrial inner segments and myopia‑associated microenvironmental changes may also contribute to photoreceptor impairment.^50^^–^^53^ In the present study, we observed a significant positive correlation between the proportion of dark cells and AL. Although the precise mechanisms underlying this phenomenon remain to be fully elucidated, we propose a hypothesis, hoping these findings offer a novel perspective on the structural alterations accompanying myopic progression. Future longitudinal studies using multimodal adaptive optics imaging will be essential to clarify whether these hyporeflective photoreceptors result from outer segment tilting, and to distinguish between reversible adaptive responses and irreversible degenerative changes. Such insights may offer novel therapeutic targets and evidence for early myopia intervention.

Beyond photoreceptor alterations, our study is the first to report nerve fiber-level changes associated with myopia progression in a pediatric group using AO-SLO. Prior OCT-based adult studies have consistently shown that myopia is associated with thinning of the peripapillary retinal nerve fiber layer, yet such observations primarily reflect macroscopic loss of tissue thickness.^54^^,^^55^ By contrast, our AO-SLO data capture the lateral geometry of individual nerve fiber bundles, revealing that bundle width progressively decreases with elongating AL even in children who have not yet developed high myopia or obvious structural atrophy.

Overall, our data delineate a previously unrecognized response in which axial elongation is closely associated with ocular tissue remodeling and subsequent microstructural alterations. Specifically, concomitant with axial elongation, we observed a reduction in photoreceptor density, an increase in photoreceptor spacing, an elevated proportion of dark cones, and remodeling of the nerve fiber layer. The mechanisms underlying these changes remain unclear; nevertheless, our findings suggest that myopia is not merely an optical disorder but rather a progressive tissue-level disease, in which multiple retinal layers are affected even at early stages. The ability of AO-SLO to detect such microstructural alterations before they become clinically manifest underscores its potential utility in risk stratification and individualized intervention planning.

Notably, the effect of AL on retinal microstructural parameters remained significant after adjusting for image quality (confidence score), thereby accounting for potential measurement bias related to deteriorated image quality in longer eyes. This finding further supports that axial elongation is an independent risk factor for photoreceptor changes.

In summary, our study identifies a constellation of myopia-associated retinal microstructural changes, encompassing decreased photoreceptor packing density, widened intercone spacing, increased prevalence of dark cones, and remodeling of the nerve fiber layer. These findings collectively advance our understanding of the underlying mechanisms of myopia and provide a potential basis for explaining clinically relevant symptoms such as decreased visual acuity. More importantly, these microstructural metrics may function as early warning biomarkers for fast progression, high-risk phenotypes, and pathological myopia, offering valuable guidance for personalized clinical decision making and early intervention.

We acknowledge that this study has several inherent limitations. The most critical is its cross‑sectional design, which precludes any conclusion regarding causality or the temporal relationship between axial elongation and retinal microstructural changes. Consequently, we are unable to determine whether the alterations in dark cells and nerve fiber width reflect reversible adaptive responses to mechanical stress or the earliest manifestations of irreversible degenerative processes. Additionally, our outcome assessment was confined to refractive error progression, without evaluating functional correlates such as visual acuity; therefore, the functional significance of increased dark cell proportion remains to be established. Moreover, the Littmann correction applied in this study was based solely on AL, as corneal curvature and anterior chamber depth could not be included due to practical constraints. This limitation may have introduced residual magnification error, and it is also well documented that imaging quality deteriorates with increasing AL, potentially affecting the accuracy of photoreceptor quantification. These challenges are inherent to studies involving highly myopic eyes and should be interpreted with caution.

## Conclusions

These findings demonstrate that axial elongation is accompanied by a series of cellular‑level alterations in retinal microstructure. These changes not only provide new insights into the pathogenesis and progression of myopia, but also reveal a potential structural basis that could serve as an early intervention target. Furthermore, as a noninvasive, high‑resolution imaging modality, AO-SLO exhibits unique clinical translational potential for the early warning and large‑scale screening of myopia, offering a novel technological pathway for individualized prediction and precise prevention of myopia.

## Supplementary Material

Supplement 1
