# Supplementary material for: Associations of Axial Length With Photoreceptor Mosaic and Retinal Nerve Fiber Bundle Microstructure Assessed by AO-SLO in Children
Source: Invest Ophthalmol Vis Sci. 2026 Oct 21;67(12):40. doi: 10.1167/iovs.67.12.40 (PMC13712400; doi:10.1167/iovs.67.12.40)
Supplement: Supplementary file 1 [file iovs-67-12-40_s001.docx]

**Supplementary Figure S1. Multiple linear regression of AO-SLO-derived parameters with AL.**

**Supplementary Figure S2. Multiple linear regression of AL and microstructural changes**

(A) Multiple linear regression of AL and dark cell ratio. (B) Multiple linear regression of AL and width of never fiber. (C) Multiple linear regression of dark cell ratio and width of dark cell ratio.

|  | Parameters | Group A | Group B | Group C | Group D | *F* | *P* Value |
| --- | --- | --- | --- | --- | --- | --- | --- |
| Temporal | PCPD, mm^2^ | 38,015.04 ± 5,262.87 | 35,625.68 ± 5,650.16 | 34,789.40 ± 5,204.59 | 31,349.04 ± 4,823.28 | 8.027 | < 0.001 |
|  | Cell spacing, μm | 4.33 ± 0.28 | 4.48 ± 0.28 | 4.52 ± 0.26 | 4.67 ± 0.27 | 7.629 | < 0.001 |
|  | Regularity (%) | 96.47 ± 1.53 | 96.67 ± 1.43 | 96.24 ± 1.35 | 96.30 ± 1.30 | 0.627 | 0.599 |
|  | Dispersion (%) | 14.29 ± 2.24 | 14.56 ± 2.82 | 14.62 ± 2.41 | 15.56 ± 2.51 | 1.483 | 0.222 |
|  | N < 4 (%) | 0.01 ± 0.05 | 0.00 ± 0.00 | 0.00 ± 0.08 | 0.00 ± 0.00 | 1.212 | 0.308 |
|  | N = 4 (%) | 1.66 ± 1.14 | 2.24 ± 1.26 | 1.96 ± 1.10 | 1.95 ± 0.82 | 1.360 | 0.258 |
|  | N = 5 (%) | 25.02 ± 2.21 | 23.62 ± 3.02 | 24.60 ± 3.15 | 26.86 ± 4.89 | 4.768 | 0.004 |
|  | N = 6 (%) | 48.93 ± 5.23 | 49.84 ± 6.87 | 48.74 ± 6.16 | 45.75 ± 4.67 | 2.916 | 0.037 |
|  | N = 7 (%) | 20.79 ± 2.32 | 20.97 ± 2.72 | 21.06 ± 2.34 | 22.08 ± 1.76 | 1.962 | 0.123 |
|  | N = 8 (%) | 3.30 ± 1.39 | 3.05 ± 1.27 | 3.45 ± 1.27 | 3.47 ± 1.12 | 0.789 | 0.502 |
|  | N > 8 (%) | 0.26 ± 0.32 | 0.28 ± 0.40 | 0.26 ± 0.33 | 0.36 ± 0.34 | 0.655 | 0.582 |
| Nasal | PCPD, mm^2^ | 37,404.75 ± 4,187.33 | 36,836.57 ± 5,773.14 | 35,649.29 ± 6,698.26 | 32,538.82 ± 8,462.83 | 3.280 | 0.023 |
|  | Cell spacing, μm | 4.35 ± 0.22 | 4.42 ± 0.27 | 4.50 ± 0.27 | 4.65 ± 0.36 | 5.914 | 0.001 |
|  | Regularity (%) | 96.40 ± 1.80 | 96.76 ± 1.40 | 96.57 ± 1.75 | 95.73 ± 1.20 | 2.668 | 0.051 |
|  | Dispersion (%) | 14.68 ± 2.62 | 14.02 ± 2.90 | 14.63 ± 4.14 | 15.08 ± 3.15 | 0.534 | 0.660 |
|  | N < 4 (%) | 0.00 ± 0.01 | 0.01 ± 0.04 | 0.00 ± 0.02 | 0.01 ± 0.04 | 0.374 | 0.772 |
|  | N = 4 (%) | 2.24 ± 1.51 | 1.91 ± 1.24 | 2.11 ± 1.60 | 2.35 ± 1.42 | 0.519 | 0.670 |
|  | N = 5 (%) | 24.83 ± 4.11 | 23.99 ± 2.82 | 24.86 ± 3.93 | 25.21 ± 3.88 | 0.608 | 0.611 |
|  | N = 6 (%) | 49.81 ± 7.92 | 50.23 ± 7.02 | 48.30 ± 10.06 | 47.53 ± 7.44 | 0.717 | 0.544 |
|  | N = 7 (%) | 20.30 ± 2.32 | 20.62 ± 2.69 | 20.87 ± 4.21 | 20.70 ± 4.36 | 0.131 | 0.941 |
|  | N = 8 (%) | 3.37 ± 1.52 | 2.96 ± 1.30 | 3.68 ± 2.35 | 3.72 ± 1.46 | 1.286 | 0.282 |
|  | N > 8 (%) | 0.35 ± 0.39 | 0.28 ± 0.28 | 0.29 ± 0.38 | 0.34 ± 0.31 | 0.328 | 0.805 |
| Superior | PCPD, mm^2^ | 36,887.69 ± 4,094.67 | 33,689.26 ± 3,627.25 | 33,660.69 ± 4,254.17 | 31,591.74 ± 3,335.26 | 8.878 | < 0.001 |
|  | Cell spacing, μm | 4.47 ± 0.27 | 4.66 ± 0.27 | 4.63 ± 0.21 | 4.75 ± 0.24 | 6.577 | < 0.001 |
|  | Regularity (%) | 97.05 ± 1.38 | 96.81 ± 1.82 | 96.98 ± 1.49 | 96.57 ± 1.26 | 0.616 | 0.606 |
|  | Dispersion (%) | 13.47 ± 2.39 | 13.85 ± 3.01 | 14.33 ± 2.82 | 14.30 ± 2.37 | 0.676 | 0.569 |
|  | N < 4 (%) | 0.00 ± 0.00 | 0.00 ± 0.00 | 0.00 ± 0.00 | 0.00 ± 0.00 | 0 | 0 |
|  | N = 4 (%) | 1.44 ± 0.92 | 1.60 ± 1.22 | 1.55 ± 0.98 | 1.83 ± 0.96 | 0.764 | 0.517 |
|  | N = 5 (%) | 23.55 ± 3.02 | 23.39 ± 2.88 | 24.28 ± 2.51 | 24.85 ± 2.185 | 2.028 | 0.114 |
|  | N = 6(%) | 52.16 ± 6.31 | 51.40 ± 6.85 | 50.17 ± 5.38 | 48.49 ± 4.50 | 2.274 | 0.083 |
|  | N = 7 (%) | 20.01 ± 2.38 | 20.40 ± 2.04 | 21.00 ± 1.85 | 21.47 ± 1.71 | 3.112 | 0.029 |
|  | N = 8 (%) | 2.71 ± 1.21 | 2.96 ± 1.61 | 2.85 ± 1.38 | 3.17 ± 1.13 | 0.604 | 0.614 |
|  | N > 8 (%) | 0.27 ± 0.32 | 0.17 ± 0.33 | 0.19 ± 0.26 | 0.23 ± 0.31 | 0.584 | 0.626 |
| Inferior | PCPD, mm^2^ | 37,026.30 ± 4,918.90 | 35,511.45 ± 5,956.32 | 34,514.57 ± 5,470.57 | 32,012.48 ± 4,059.36 | 4.828 | 0.003 |
|  | Cell spacing, μm | 4.40 ± 0.37 | 4.46 ± 0.28 | 4.56 ± 0.33 | 4.68 ± 0.28 | 4.538 | 0.005 |
|  | Regularity (%) | 96.27 ± 1.35 | 96.51 ± 1.49 | 96.38 ± 1.32 | 96.26 ± 1.18 | 0.235 | 0.872 |
|  | Dispersion (%) | 14.82 ± 1.93 | 14.73 ± 2.65 | 14.16 ± 2.21 | 14.63 ± 1.85 | 0.608 | 0.611 |
|  | N < 4 (%) | 0.00 ± 0.00 | 0.01 ± 0.05 | 0.03 ± 0.09 | 0.00 ± 0.02 | 1.759 | 0.159 |
|  | N = 4 (%) | 1.95 ± 0.92 | 2.14 ± 1.35 | 2.03 ± 1.11 | 2.27 ± 0.76 | 0.500 | 0.683 |
|  | N = 5 (%) | 24.73 ± 2.54 | 24.67 ± 3.06 | 23.90 ± 2.28 | 25.12 ± 2.40 | 1.394 | 0.248 |
|  | N = 6 (%) | 49.05 ± 4.87 | 47.94 ± 6.32 | 49.48 ± 5.60 | 46.76 ± 4.12 | 1.729 | 0.164 |
|  | N = 7 (%) | 20.63 ± 2.00 | 21.77 ± 1.96 | 20.97 ± 2.13 | 22.15 ± 2.02 | 3.505 | 0.018 |
|  | N = 8 (%) | 3.40 ± 1.26 | 3.23 ± 1.37 | 3.44 ± 1.18 | 3.44 ± 1.06 | 0.215 | 0.886 |
|  | N > 8 (%) | 0.30 ± 0.27 | 0.25 ± 0.34 | 0.17 ± 0.34 | 0.30 ± 0.37 | 10143 | 0.335 |

**Supplementary Table S1. Summary of AO-SLO-derived imaging parameter.**

| Group | Variables | β (95% CI) | *P* Value | VIF |
| --- | --- | --- | --- | --- |
| PCPD | | | | |
| Temporal | AL, mm | -3154 (-3864, -2445) | < 0.0001 | 1.003 |
|  | Confidence (%) | 260.6 (142.1, 379.0) | < 0.0001 | 1.003 |
| Nasal | AL, mm | -2918 (-3653, -2182) | < 0.0001 | 1.017 |
|  | Confidence (%) | 362.3 (233.8, 490.8) | < 0.0001 | 1.017 |
| Superior | AL, mm | -2110 (-2767, -1454) | < 0.0001 | 1.053 |
|  | Confidence (%) | 56.58 (-38.22, 151.4) | 0.2396 | 1.053 |
| Inferior | AL, mm | -2847 (-3596, -2098) | < 0.0001 | 1.017 |
|  | Confidence (%) | 248.9 (112.7, 385.0) | 0.0004 | 1.017 |
| Cell spacing | | | | |
| Temporal | AL, mm | 0.1926 (0.1548, 0.2304) | < 0.0001 | 1.003 |
|  | Confidence (%) | -0.004028 (-0.01035, 0.002290) | 0.2092 | 1.003 |
| Nasal | AL, mm | 0.1639 (0.1228, 0.2051) | < 0.0001 | 1.015 |
|  | Confidence (%) | -0.0009648 (-0.01684, 0.002459) | 0.0090 | 1.015 |
| Superior | AL, mm | 0.1365 (0.09682, 0.1761) | < 0.0001 | 1.053 |
|  | Confidence (%) | 0.01165 (0.005918, 0.01738) | 0.0001 | 1.053 |
| Inferior | AL, mm | 0.1821 (0.1375, 0.2268) | < 0.0001 | 1.017 |
|  | Confidence (%) | -0.001436 (-0.009551, 0.006678) | 0.7266 | 1.017 |

**Supplementary Table S2. Multiple linear regression of AO-SLO-derived parameters with AL.**

| Variables | β (95% CI) | *P* Value | VIF |
| --- | --- | --- | --- |
| AL, mm | 0.7887 (0.4121, 1.165) | < 0.0001 | 1.003 |
| Confidence (%) | 0.09893 (0.03628, 0.1616) | 0.0022 | 1.003 |

**Supplementary Table S3. Multiple linear regression of AL and dark cell ratio.**

| Variables | β (95% CI) | *P* Value | VIF |
| --- | --- | --- | --- |
| AL, mm | -7.439 (-9.059, -5.819) | < 0.0001 | 1.001 |
| Confidence (%) | 0.1696 (-0.1122, 0.4514) | 0.2356 | 1.001 |

**Supplementary Table S4. Multiple linear regression of AL and width of nerve fiber.**

| Variables | β (95% CI) | *P* Value | VIF |
| --- | --- | --- | --- |
| AL, mm | -7.409 (-9.166, -5.652) | < 0.0001 | 1.149 |
| Dark cell ratio (%) | -5.279 (-83.15, 72.59) | 0.8934 | 1.149 |

**Supplementary Table S5. Multiple linear regression of AL/dark cell ratio and width of nerve fiber.**
